# Using the anterior capsule of the hip joint to protect the tensor fascia lata muscle during direct anterior total hip arthroplasty: a randomized prospective trial

**DOI:** 10.1186/s12891-019-3035-9

**Published:** 2020-01-11

**Authors:** Gongyin Zhao, Ruixia Zhu, Shijie Jiang, Nanwei Xu, Hongwei Bao, Yuji Wang

**Affiliations:** 1 Department of Orthopedics, Changzhou No.2 People’s Hospital, the Affiliated Hospital of Nanjing Medical University, Changzhou, China; 2 Department of Orthopedics, Jingjiang People’s Hospital, 28, Zhongzhou East road, Taizhou, China; 3 Departments of Orthopedic Surgery and Biochemistry and Molecular Biology, Mayo Clinic, 200 First St. SW, Rochester, MN 55905 USA; 4 Department of Orthopedics, the Third Affiliated Hospital of Gansu University of Chinese Medicine, 222 Silong Road, Baiyin, 730900 China

**Keywords:** Direct anterior approach, Total hip arthroplasty, Tensor fasciae lata muscle, Capsular, Protect, Trauma, Muscle cross-sectional area, Fatty atrophy

## Abstract

**Background:**

The direct anterior approach for total hip arthroplasty (THA) has specific advantages, but injury to the tensor fasciae lata muscle (TFLM) remains a concern. This injury in part negates some of the advantages of the intermuscular approach, because injury of the muscle fibers of the TFLM can lead to less satisfactory clinical results. Thus, in this study, we propose an intraoperative method to protect the TFLM and demonstrate its feasibility.

**Methods:**

Fifty-six patients undergoing THA by the direct anterior approach were divided randomly into two groups. In group A, the TFLM was protected by an autogenous tissue “pad” created from the anterior capsule of the joint which protect the TFLM from direct contact with the retractors. In group B, the operation was carried out with no protection of the TFLM except the attempt by the surgeons to consciously avoid injury of the TFLM. We evaluated magnitude of changes in the muscle cross-sectional area (MSCA) and fatty atrophy (FA) by magnetic resonance imaging. The differences in blood hemoglobin and serum levels of myoglobin, lactate dehydrogenase (LDH), and creatine phosphokinase (CPK) were compared at different time, postoperatively. The Harris hip score, postoperative drainage volume and visual analogue scores (VAS) were compared between the two groups.

**Results:**

LDH, CPK and myoglobin in group B were significantly higher than group A at 8, 24, and 48 h after the surgery. (*p* < 0.05) Compared to the group A, the decrease of hemoglobin in group B displayed significantly at 24 and 48 h after surgery. (*P* < 0.05) The significantly increased MSCA and FA of TFLM were demonstrated in group B. The PDV and VAS in group B were significantly higher than group A. (P < 0.05) The Harris score in group A was significantly higher than group B (P < 0.05) one month after surgery, but there was no significant difference six months later.

**Conclusions:**

Using the anterior capsule of the hip joint as an autogenous, protective capsular tissue pad to limit the trauma to the TFLM during a direct anterior approach to THA is an effective method to protect the TFLM and improve the clinical effect.

**Trial registration:**

ChiCTR: ChiCTR1900025173. Retrospectively registered August 15, 2019.

## Introduction

The direct anterior approach (DAA) is considered a more minimally invasive operative approach for total hip arthroplasty (THA). This approach is favored by surgeons, because it utilizes the intermuscular plane, thereby avoiding the need to transect any of the periarticular muscles. Moreover, this approach is favored because it provides adequate exposure of the acetabular side [1–5]. Several studies have reported that DAA decreases the duration of hospital stay and increases the percentage of patients discharged home due to the lesser operative trauma which permits a more rapid recovery [5–9]. Whether DAA is actually superior to other surgical approaches, however, remains controversial. Due to some concerns of less adequate exposure caused by difficulty elevating of proximal femur, some complications can occur during the operation, such as contusion of periarticular muscles, injury to the anterolateral femoral cutaneous nerve, and fractures around the prosthesis [10–15].

By forming the lateral wall of the surgical wound, the muscle fibers of the tensor fascia lata muscle (TFLM) are often traumatized due to mechanical retraction by surgical instruments. While preparing the acetabulum for the prosthesis, the TFLM is often damaged by the handle of the acetabular reamer. In addition, muscle fibers of the TFLM can be injured by the edge of the retractor and blades of the saw during the process of elevating the proximal femur and implanting the femoral prosthesis. Therefore, injury of the TFLM is one of the most common complications of a DAA for THA. Inadvertent injury to the muscle can increase bleeding and postoperative pain, lead to abnormalities in gait, and overall poorer recovery for patients. Meanwhile, excessive stimulation of muscles causes irreversible injuries, such as compensatory thickening, hypertrophy, and lipogenesis of muscle fibers [13, 14, 16, 17]. Therefore, avoiding secondary injury to the periarticular muscles caused during THA operations via a DAA is an increasing concern for orthopedic surgeons. Some surgeons have used protective agents, such as gauze or plastic sheets, to prevent injury to the TFLM, some have used curved abdominal retractors, and others have used nothing but conscious intraoperative awareness of the potential for injury to these muscles. These methods have not proven satisfactory, however, because of their complexity and lack of effective protection of the muscle.

After the iliofemoral ligament and the anterior articular capsule be revealed in a DAA, many surgeons open the anterior capsule in a “T” or transverse fashion to expose the hip joint and later close the capsule by suture or not close the capsule after the arthroplasty; both closing or not closing the capsule are widely accepted [5, 18–22]. Although some surgeons believe that the anterior capsule is not important, we have found a new application for this structure. We hypothesized that we could protect the TFLM by using the anterior articular capsule as a barrier between the TFLM and the retractors needed for exposure of the joint for the arthroplasty.

This randomized, controlled prospective study was designed to determine if this maneuver would protect the TFLM during a DAA approach to hip arthroplasty. By examining the release of muscle-specific enzymes as a marker of the degree of muscle damage and using high-quality images obtained by magnetic resonance imaging (MRI) of the periarticular muscles to determine the muscle cross-sectional area (MCSA) and fatty atrophy (FA), we assessed whether this technique helped to minimize trauma, thereby protecting the integrity of the TFLM during the DAA to THA.

## Materials and methods

### Subjects

**Fig. 1:**
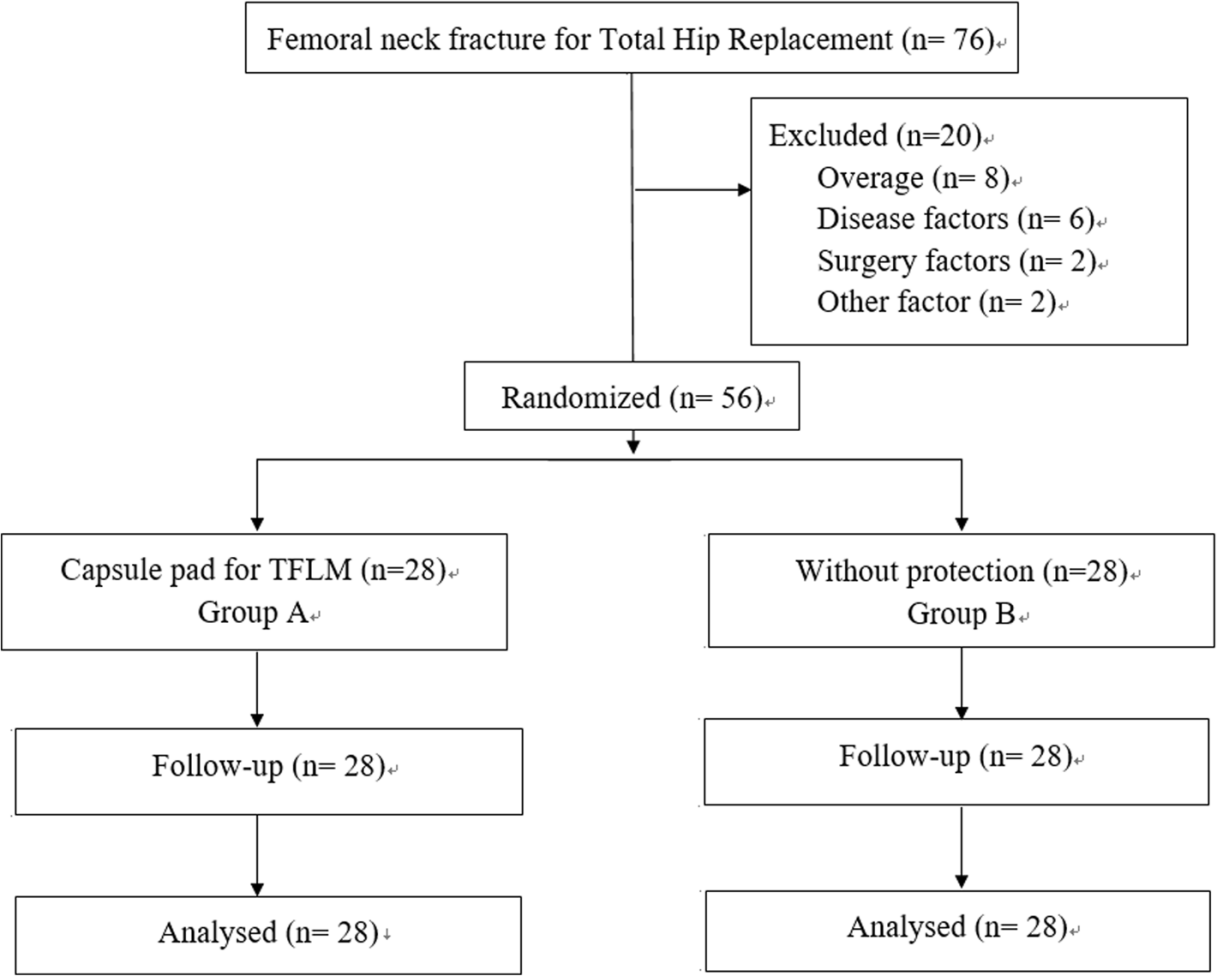
Flow diagram of patient enrollment and randomization

From March 2018 to March 2019, patients who suffered from femoral neck fracture were randomly divided into 2 groups according to the time of admission. Patients with odd day admission were involved in group A, and patients with even day were involved in group B. A supervisor nurse who did not know the surgery situation assigned patients to groups. The same group of surgeons, who had implanted more than 300 prostheses using this approach, performed all operations. Inclusion criteria: femoral neck fractures (within a week), uncemented prosthesis, THA via DAA. Exclusion criteria: advanced age (older than 75 years), hepatic or renal failure, coagulation abnormalities, thrombosis, myocardial infarction, cerebrovascular accidents (within the previous 6 months), long-term use of anticoagulants, long operation time (more than 90 min), unexpected intraoperative condition (intraoperative fracture etc.), unable to undergo an MRI (claustrophobia etc.) and could not be evaluated effectively by MRI. In the end, 56 of 76 patients were included in the study and 20 were excluded. (Fig. 1) The surgical team, consisting of three surgeons and two instrument nurses, was responsible for the surgical treatment of the two groups of patients. In Group A, the TFLM was covered by the anterior capsular pad which was rotated to cover the surface of the TFLM, while in group B, the surgeons were reminded to try their best to remember to minimize tension on, traction of the TFLM during the operation. The TFLM had no substantial protection. The approval for this study was obtained from the Institutional Review Boards of the first authors’ affiliated institutions.

**Table I:** Demographics of patients in group A and group B

	Group A	Group B	t-test	*P*-value
Age (y)	67(±5.6)	70(±5.1)	1.993	*p* > 0.05
Male (%)	10 (36%)	8 (29%)	–	–
Female (%)	18 (64%)	20 (71%)	–	–

Values are presented as means (± SD)

### Surgical technique

All patients underwent operation under general anesthesia within 5 days of femoral neck fracture. During anesthesia, the same proportion of muscle relaxants were given according to body weight. Intravenous tranexamic acid (1 g) was administered to patients in each group. All patients also received autologous blood transfusion whenever possible during the process. All operations were performed by the same group of surgeons.

The patients were placed in a supine position using a regular Table. A typical incision was made over the medial margin of the TFLM. After the location of the TFLM was confirmed, the overlying fascia was incised. After identifying the space between the TFLM and rectus femoris muscle, the TFLM was retracted laterally. After transecting the ascending branch of the lateral circumflex femoral artery, the anterior capsule was exposed satisfactorily.

On the medial side of the capsule, the iliocapsularis muscle lies posterior to the rectus femoris muscle and is attached to the anterior capsule. The iliocapsularis is an elongated muscle that is attached to the full length of the anterior capsule. Though rarely reported, some surgeons have suggested that the iliocapsularis muscle stabilizes the femoral head by contracting within the dysplastic acetabulum [19, 23, 24]. The iliocapsularis muscle serves as an anatomic marker separating the joint capsule of the hip from the hazardous area posteriorly, which contains the arteries and nerves that can be injured by operating on the superomedial aspect of the iliocapsularis muscle.

**Fig. 2:**
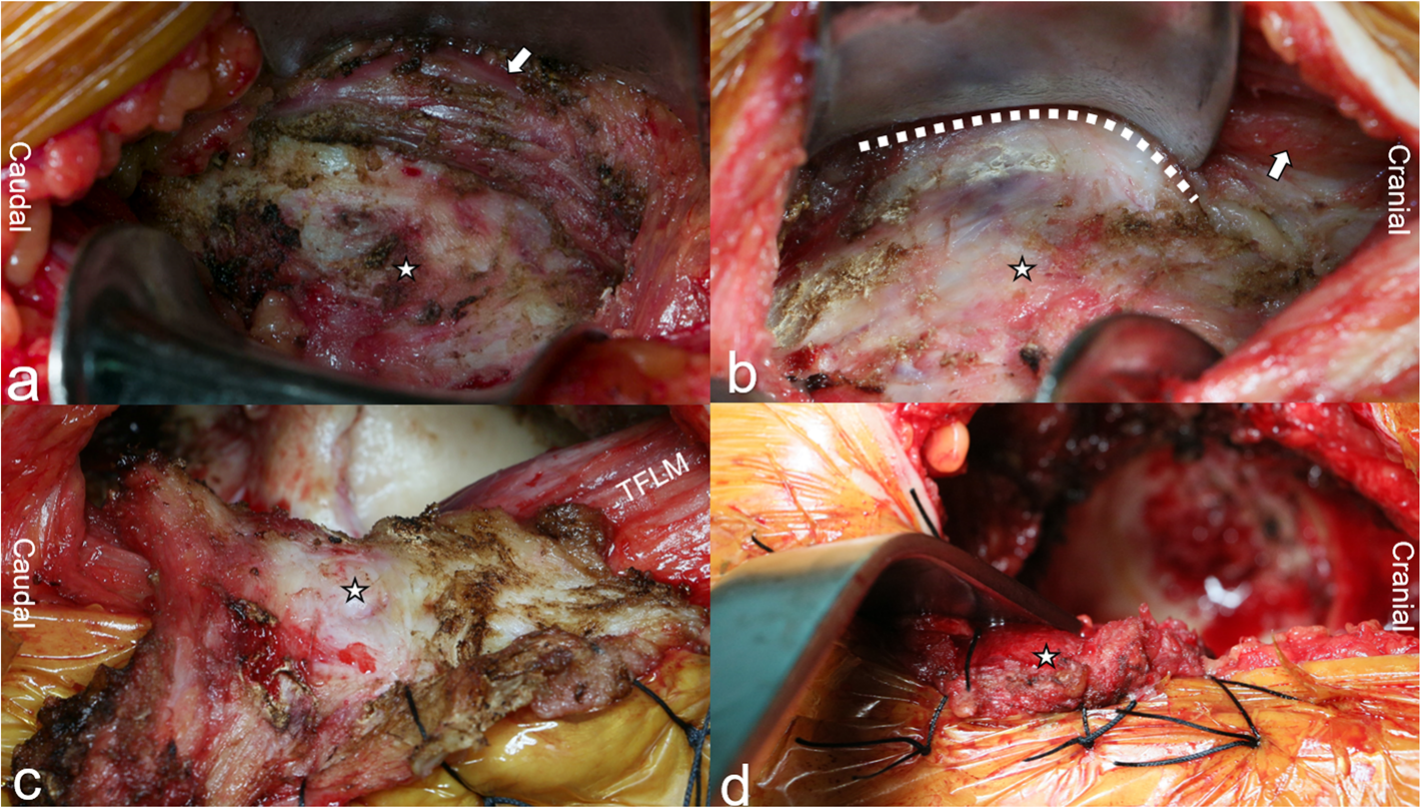
The stars represent the anterior capsule in **a** and **b**, and in **c** and **d** the stars represent the capsule pad that is prepared from the anterior capsule. The white arrows indicate the iliocapsularis, which can be separated from the capsule along the dotted line in **b**. The detached capsule is flipped over the TFLM in **c**. The capsule pad is spaced between the retractor and the TFLM in **d**

**Fig. 3:**
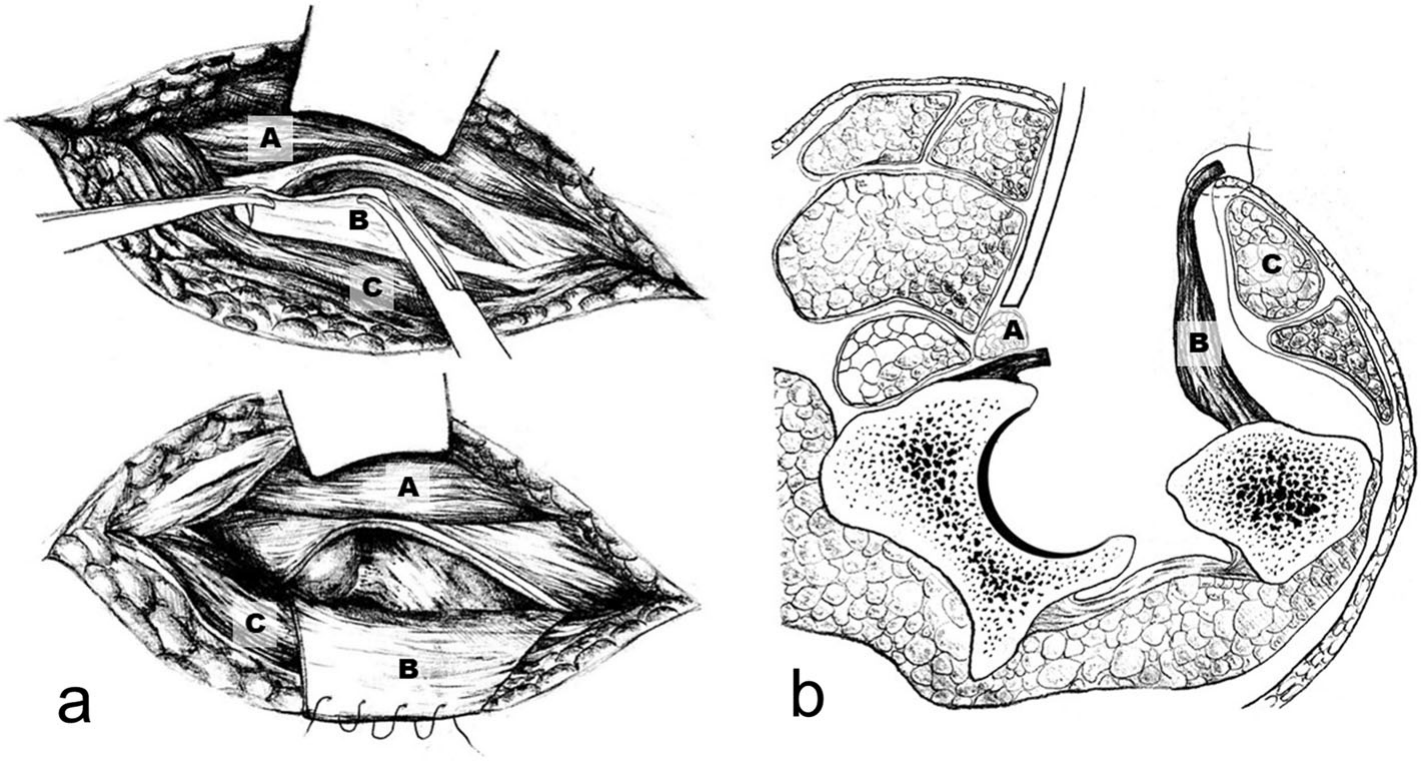
Figure a is the anatomy diagram of the capsule tissue pad, and figure b is the section diagram. **a** represents the iliocapsularis, **b** represents the anterior capsule, **c** indicates the TFLM

Keeping the iliocapsularis muscle as the medial boundary of the joint capsule, we dissected along the lateral side of the iliocapsularis, being careful to detach the muscle from the joint capsule to expose the maximum possible area of the anterior articular capsule. After incising the articular capsule, the now separated anterior capsule was folded outward as a layer of soft tissue acting as a “pad” covering the outer musculature and sutured to the skin at the edge of the incision with silk suture material. With this maneuver, the retractor and the handle of the reamer were separated by the “capsular pad” from direct contact with the TFLM, while still allowing the exposure of the hip joint for the THA. The specific steps are described in (Fig. 2 and Fig. 3).

In group B, the anterior capsule was incised in the middle and retracted to the sides to expose the hip joint. The TFLM was protected only by the surgeon’s conscious effort to avoid undue trauma to the muscle during the exposure of the joint to allow adequate visualization for the THA, without the use of special instruments or devices.

In both groups, acetabular and femoral prostheses were implanted in an identical way. Each patient received a non-cemented femoral stem, a press-fit acetabular cup, and a 32-mm ceramic head and ceramic lining. The prosthesis came from a single manufacturer (Waldemar Link Gmbh & Co, Hamburg, Germany). The capsule was then re-sutured closed after the arthroplasty was completed. No other structures were injured during the operation, and all 56 patients had their THA completed within 90 min. Drainage tubes were routinely placed after the operation to allow wound drainage for 48 h. Antibiotics were administered perioperatively for 48 h, and nadroparin calcium as prophylaxis against deep vein thrombosis was administered within 6 h postoperatively. After the patients were fully wake, they were encouraged to walk immediately with the aid of a walker.

At 8, 24, and 48 h after surgery, blood hemoglobin (Hb), serum levels of creatine kinase (CK), lactate dehydrogenase (LDH), and myoglobin (MYO), as well as postoperative drainage from the wound drains [13], were recorded and later compared between the two groups. In addition, we used a VAS scale to score pain at 8, 24, and 48 h after surgery.

No patient developed infection, facture, or other complications, and none of the patients in ether group required any allogeneic blood transfusions during the perioperative period. All patients were discharged from the hospital within one week, with the shortest discharge 3 days after surgery and the longest 7 days after the THA. Patients were required to return to the hospital once a month in the first six months after surgery and Harris scores were calculated in each visit. Harris scores at 1 and 6 months after surgery were recorded and compared between the two groups. A single observer blinded to the patient group collected the relevant clinical data.

**Fig. 4:**
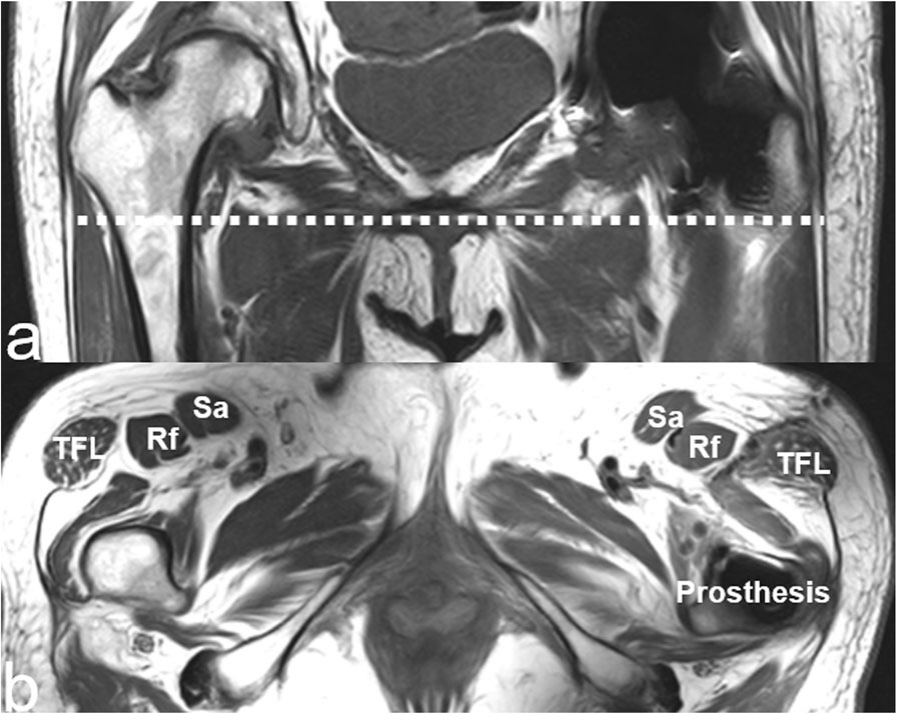
**a** and **b** show typical measurements of CSA and fatty atrophy of the TFLM before and after surgery in group **a**. Measurements for group **b** are shown in **c** and **d**. Photographs of the TFLM after arthroplasty corresponding to the left MRI image are shown in **e** and **f**. The TFLM was protected with the capsule pad as shown in **c**. In group **b**, evidence of substantial damage to the TFLM is shown

An MRI was obtained 4 weeks after surgery and compared with the preoperative MRI. Imaging was performed on a 3.0-T MR tomograph (Achieva 3.0 T TX, Philips, Netherlands) according to a standard protocol. The T2-weighted, cross-sectional images were obtained at the level of the lesser trochanter. The location of the MRI cross-section is shown in (Fig. 4). By fully identifying the surrounding muscle tissue, the TFLM was located and its cross-sectional area and degree of fatty atrophy were measured. The TFLM was identified in the images by its elliptical shape and its position anterolateral to the femur and lateral to the rectus femoris muscle. As a measure of pathologic change, fatty atrophy was first identified and classified by Daniel in 1994. The classification system quantitated fatty changes in the rotator cuff into 5 grades: Grade 0, no fat; Grade 1, scant amounts of fat; Grade 2, fat visible but less than muscle; Grade 3, muscle and fat in equal amounts; and Grade 4, fatty streaks present more than muscle. Florian Engelken modified the classification to give the percentage of fatty tissue increments of 25% in 2014 [25, 26]. We adopted the classification method of Daniel et al. and divided the fatty atrophy into 5 grades. The ellipse of the TFLM was measured along its long axis and short axis to obtain the muscle cross-sectional area from the formula.

### Analysis of data

In order to determine the effects of the two types of operative attempts to protect the TFLM, we measured the mean level of blood Hb and serum muscle-specific enzymes, as well as the mean changes in fatty atrophy and cross-sectional areas, within groups before and after the THA. For wound drainage, because the data were not normally distributed, we calculated the median drainage (with interquartile ranges as well as actual ranges). All images were analyzed by an observer who was blinded to both groups.

### Statistical analysis

We used a Student’s paired *t* test to compare the differences in the clinical and imaging data with or without use of the capsular pad and a Kruskal Wallace test for wound drainage. *P* values < 0.05 were considered statistically significant. The data were analyzed by GraphPad Prism 5 software.

## Results

**Fig. 5:**
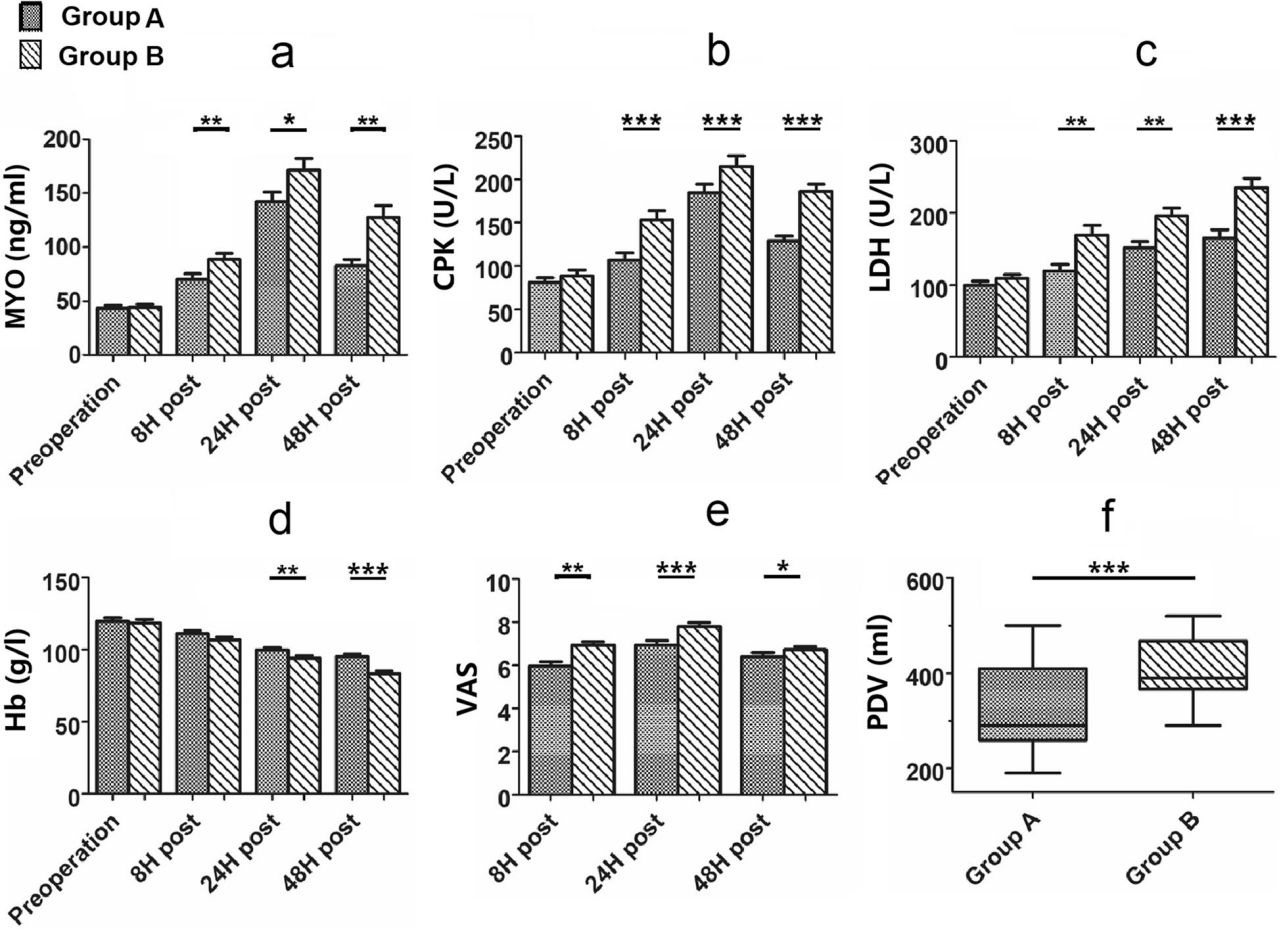
Comparison of laboratory examination results, postoperative pain scores, and postoperative drainage volumes between the two groups of patients at pre-operation and three time periods post-operation. * *p* < 0.05, ** *p* < 0.01, ***p < 0.001

There were no statistically significant differences in the demographics or the results of preoperative laboratory examinations between the two groups. The BMI of the two groups was similar. Baseline demographic information of the patients is shown in Table 1, which confirms the comparability of the patients. In contrast, at 8, 24, and 48 h postoperatively, serum levels of MYO, CPK, and LDH were significantly greater in group B than in group A (Fig. 5a, b, c; *p* < 0.05 each). When evaluating the decrease in blood Hb at 8 h postoperatively, there was a trend in group B having a greater decrease in Hb content (*p* = 0.06); however, the decrease in blood Hb became greater in group B at 24 and 48 h postoperatively (Fig. 5d, *p* < 0.01).

When evaluating postoperative pain, the VAS scores were higher in group B than in group A at 8, 24 and 48 h postoperatively (Fig. 5e; *p* < 0.01, p < 0.01, 0.01 < p < 0.05). The amount of wound drainage in group A was also less than in group B (Fig. 5f; p < 0.01). These results are summarized in (Fig. 5).

**Fig. 6:**
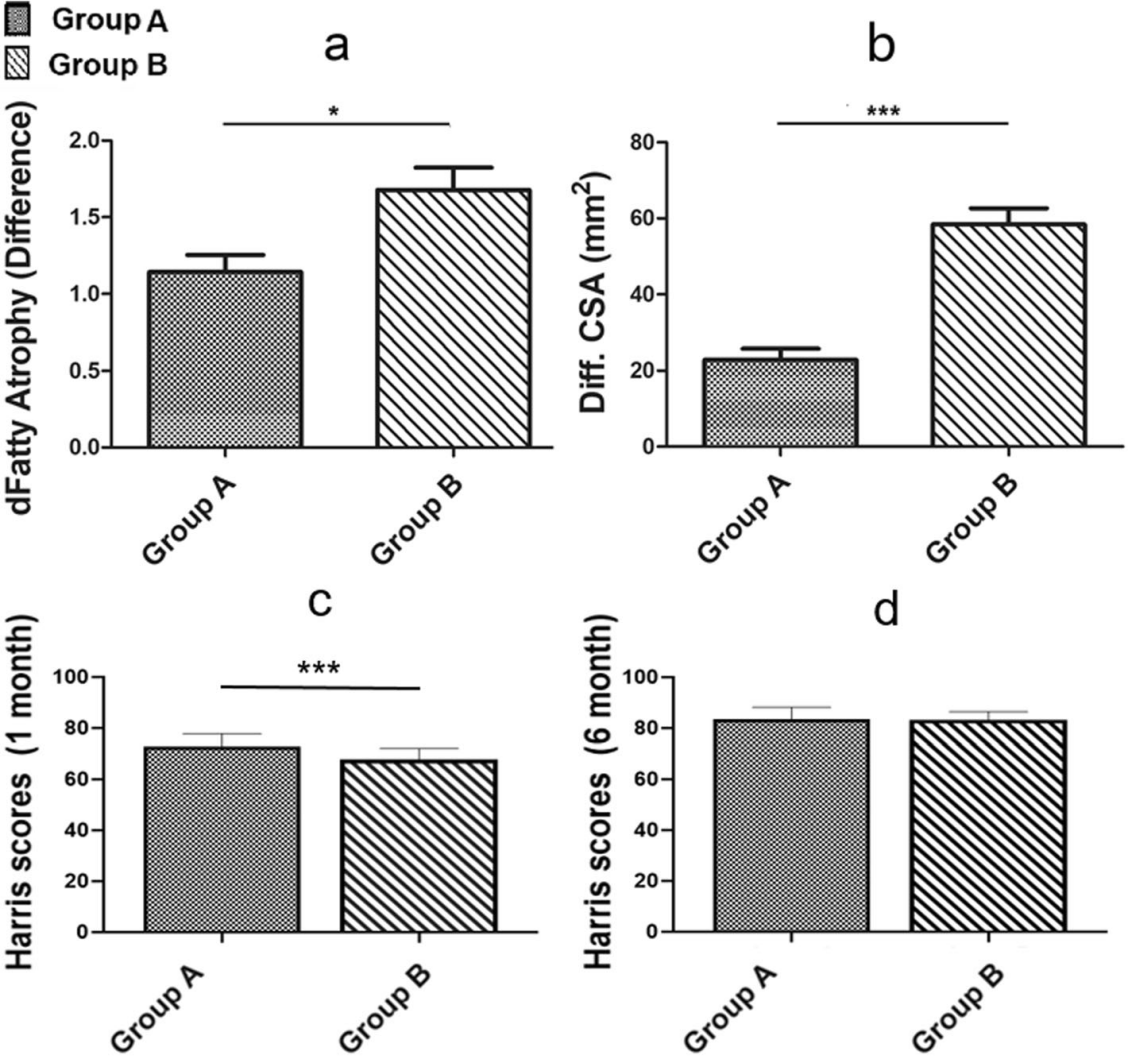
Fig. **a** demonstrates the ‘Fatty Atrophy’ difference in grades of fatty atrophy before and after operation. Fig. **b** indicates the ‘CSA’ difference between preoperative and postoperative CSA of TFLM. Fig. **c** shows the Harris scores of the two groups 1 month after surgery. Fig. **d** shows the Harris scores of the two groups 6 month after surgery. *, * p < 0.05, ** p < 0.01, ***p < 0.001

**Fig. 7:**
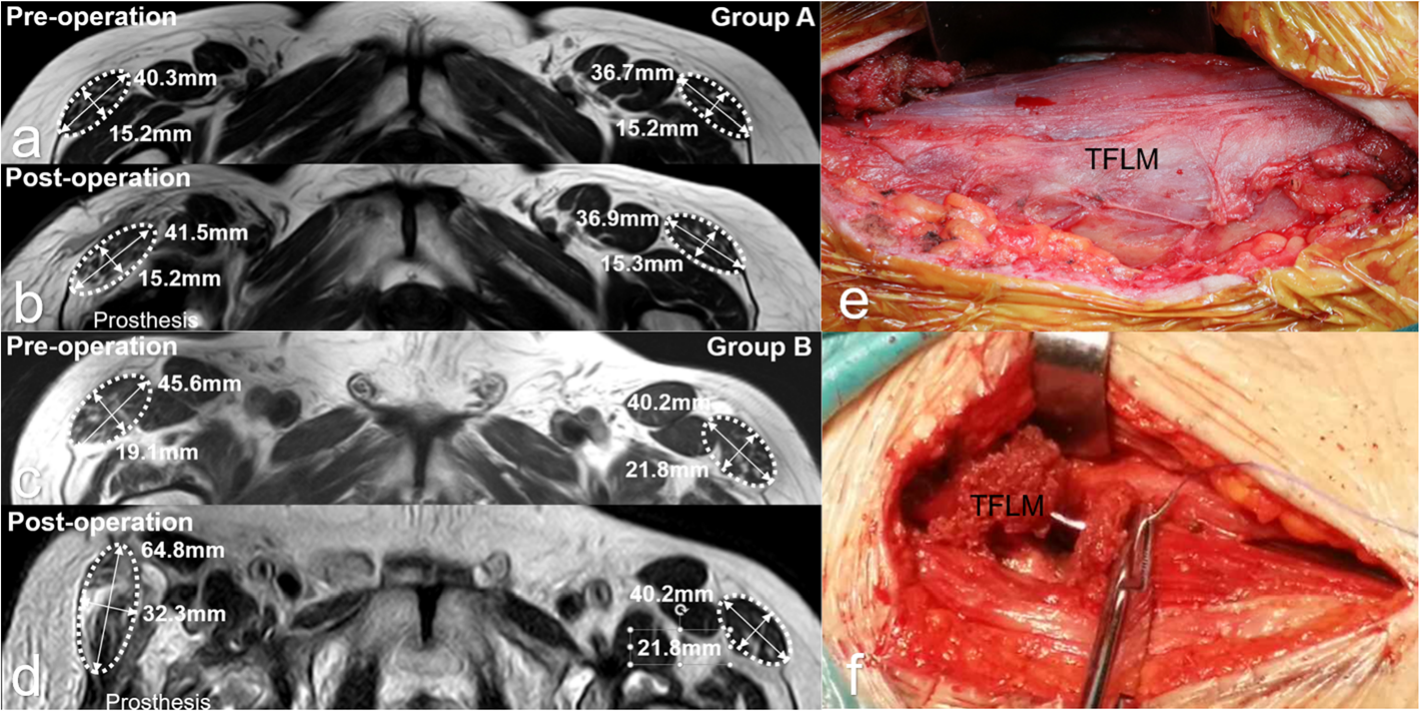
The measured plane we selected is shown with the dotted line on the coronal position in **a**. **b** shows anatomic features before measurement. TFL indicates the TFLM, Rf represents the rectus femoris, and Sa represents the rectus femoris sartorius

According to the comparative analysis of the MRI imaging data from the two groups, the degree of fatty atrophy of the TFLM as a measure of muscle injury was significantly less in group A than that of group B (Fig. 6a; p < 0.01), as was the volume of the TFLM cross sectional area (Fig. 6b; p < 0.01). Patients in group A had the significantly higher Harris hip scores 1 month after the surgery (Fig. 6c; p < 0.01). However, six months after surgery, the Harris scores were similar in both groups (Fig. 6d). These observations and measurements of the MRI imaging were shown in (Fig. 7).

## Discussion

The intermuscular plane is utilized in the DAA for THA; however, due to the more limited exposure of the hip joint, in many cases the necessary retraction of the TFLM to expose the joint adequately to allow a safe and reliable THA can lead to potentially serious tissue injury of the periarticular muscles, including the TFLM, gluteus medius, and rectus femoris muscles. This type of muscle injury can lead to more postoperative pain and delayed physical rehabilitation [2, 3, 27]. We used the anterior capsule as a protective autogenous “capsular tissue pad” to try to minimize collateral damage, especially to the TFLM during the operation. While this maneuver cannot protect all of the periarticular tissues that might be traumatized during a THA, the use of this autogenous capsular tissue pad appears to protect the TFLM substantially.

Using nearby tissues to protect important structures is a common practice in surgery. The key point in using the capsular tissue pad is to identify the iliocapsularis muscle and transect the anteromedial capsule along the length of the iliocapsularis muscle, thereby exposing the largest area of articular tissue pad. The iliocapsularis, which is attached to the anterolateral aspect of the capsule, is rarely mentioned in the anatomy of the hip. In this study, we show what we believe to be an important role of the iliocapsularis muscle [19, 23, 24, 28–30].

We used objective measures of hematologic parameters of blood loss, serum levels of circulating, muscle-specific enzymes, and quantitative MRI imaging to determine the extent of muscle damage. As indicators of muscle damage, MYO, CPK, and LDH were used to determine the extent of cell muscle injury [14, 17, 31, 32]. Among these data, CPK showed the greatest relative differences in the three postoperative time periods, while the differences in LDH and MYO were less impressive though still statistically significant. We postulate that these somewhat delayed changes in group B are related to the time sequence of anabolism and catabolism of some proteins. Alterations in Hb indicate the amount of blood lost, which can indicate soft tissue damage indirectly. The decrease in hemoglobin became more pronounced and the difference between the two groups more obvious over time, which may be related to “invisible blood loss” into the periarticular tissues after the THA. Consistent with this delay was the lack of a statistically significant difference in the VAS pain scores at 8 h postoperatively; however, the difference became statistically significant at 24 and 48 h postoperatively. These findings suggest that using the capsular tissue pad can improve patient experience. The absence of effective protection of the TFLM results in damage to the muscle, which can cause more postoperative bleeding, increase postoperative drainage, and may also cause a delayed recovery during rehabilitation.

MRI was used to visualize muscle injury. Comparing the two groups of patients, we found that the fatty atrophy was more severe in the group not protected with the capsular tissue pad. In addition, the CSA of TFLM became larger, indicative of muscular edema and possibly inflammation. Some studies have mentioned that there is increased fatty atrophy with greater injury, associated with a decrease in muscle CSA [14, 25, 33]. We believe the different timing of post-operative MRI examinations causes this inconsistency. When a muscle is damaged, it usually goes through three stages: the destruction and inflammatory phase (1 to 3 days), the repair phase (3 to 4 weeks), and the remodeling phase (3 to 6 months). The edema was usually more obvious in the second stage [34–36]. We performed our MRIs four weeks after THA, but according the relevant literature, the earliest reports of evaluation of hip CSA were 3 months after surgery; this is probably after the edema we noted had resolved, followed by evidence of muscle atrophy.

Although the TFLM was not as important as the gluteus medius muscle in maintaining hip stability and function, the pain associated with injury affects hip joint movement and function [14, 33]. The Harris score of the patients receiving articular capsule protection was significantly higher than that of the non-protective group one month after surgery, indicating the articular capsule was effective in protecting TFLM. Nevertheless, interestingly, we found no statistically significant difference in Harris scores between the two groups six months after surgery, which we think might be due to two factors: first, scar repair of the TFLM itself; and second, hip function where the TFLM was partially compensated by other muscles.

Although both groups of patients underwent surgery under general anesthesia, anesthesiologists were not constant. Different anesthesiologists had different habits in the use of muscle relaxants, so the degree of muscle relaxation during surgery could not be unified, which may cause a certain degree of biases in the experimental results.

The advantage of this approach is that the capsular tissue pad does not require extra preparation and has enough bulk and strength to protect the muscle from the retractor and instruments used for the THA. The synovial layer of the capsule is smooth and moist, and the fibrous layer is dense and firm. As the liner of the joint, the capsule pad is both convenient and strong. After the detached articular capsule was reversed and stitched to the lateral skin, the hip joint was exposed more sufficiently. This method has its limitations. Rather than simply cutting open or excising the capsule, the need to strip carefully the capsule from its attachment to the iliocapsularis muscle requires more manipulation and takes more time. Because of the small number of patients in this study and the short follow-up time, the long-term effects of such methods need to be further observed. Another limitation of this method is that because of the size and position of the released anterior capsule, the proximal wound cannot be covered by the capsular tissue pad, and therefore the gluteus minimums and gluteus mediums muscles cannot be protected. Finally, whether too much dissection of the iliocapuslaris could lead to severe consequences still needs to be determined.

## Conclusions

In conclusion, the use of a “protective “articular capsulular tissue pad effectively decreased operative injury to the TFLM, decreased bleeding and postoperative pain.

## Supplementary information

**Additional file 1.** Consort Check list

## Data Availability

The datasets used and/or analysed during the current study are available from the corresponding author on reasonable request.

## Abbreviations

CPK: Creatine phosphokinase
DAA: Direct anterior approach
FA: Fatty atrophy
Hb: Hemoglobin
LDH: Lactate dehydrogenase
MRI: Magnetic resonance imaging
MSCA: Muscle cross-sectional area;
MYO: Myoglobin
TFLM: Tensor fasciae lata muscle
THA: Total hip arthroplasty
VAS: Visual analogue scores
